# DNA methyltransferase inhibitors upregulate CD38 protein expression and enhance daratumumab efficacy in multiple myeloma

**DOI:** 10.1038/s41375-019-0587-5

**Published:** 2019-10-08

**Authors:** Priya Choudhry, Margarette C. Mariano, Huimin Geng, Thomas G. Martin, Jeffrey L. Wolf, Sandy W. Wong, Nina Shah, Arun P. Wiita

**Affiliations:** 1 Department of Laboratory Medicine, University of California, San Francisco, CA USA; 2 Division of Hematology/Oncology, Department of Medicine, University of California, San Francisco, CA USA; 3 Helen Diller Family Comprehensive Cancer Center, University of California, San Francisco, CA USA

**Keywords:** Myeloma, Cancer immunotherapy, Cancer therapeutic resistance, Immunotherapy

## To the Editor:

Plasma cells, the malignant cells in multiple myeloma (MM), express high levels of CD38. Daratumumab, the first CD38-targeting therapeutic antibody, is well tolerated and is effective both as monotherapy and in combination regimens. Daratumumab’s efficacy in cell lines, in ex vivo patient samples, and in the clinic correlates with expression of CD38 on pretreated myeloma cells [1, 2]. There is a rapid but reversible loss of cell surface CD38 expression on MM cells during daratumumab treatment that favors immune escape/resistance and disease progression [2, 3]. These observations suggest that an increase in CD38 cell surface expression on MM cells could improve daratumumab efficacy and reverse daratumumab resistance. Therefore, efforts are underway to study the regulation of CD38 in MM. Recently, two small molecules that upregulate CD38 expression and work synergistically with daratumumab have been identified: all trans retinoic acid (ATRA) and the histone deacetylase inhibitor Panobinostat [1, 4].

Recent studies have shown that DNA methylation patterns change during MM progression, and clinically aggressive subtypes such as plasma cell leukemias and translocation t(4:14) have hypermethylation [5, 6]. DNA hypermethylation has been associated with heterochromatin formation and inactivation of tumor suppressor genes [7]. DNA methyltransferases (DNMTs) methylate cytosine residues in CpG islands and remodel chromatin. Currently two DNMT inhibitors (DNMTis) have been approved for treatment of acute myeloid leukemia and myelodysplastic syndrome: azacytidine (AZA) and decitabine (DEC). These cytidine analogs incorporate into the genome of proliferating cells during DNA synthesis, covalently bind DNMTs, targeting them for proteasomal degradation, and causing passive loss of cytosine methylation in daughter cells [7]. Based on analysis of publicly-available ENCODE data, we found a CpG island in the first exon of *CD38* and hypothesized that DNA methylation represses *CD38* expression (Fig. S1). *CD38* was previously identified as a differentially methylated region in myeloma patient samples with a negative correlation between gene expression and DNA methylation [8]. We further performed additional analyses of DNA methylation and gene expression data in three independent MM datasets (GSE43860, GSE17306, and GSE12453) and found an inverse correlation between *CD38* promoter methylation status and gene expression between normal plasma cells and malignant plasma cells in MM (Fig. S1b, c). These findings suggested that DNMTs may regulate *CD38* transcription. Here, we demonstrate that DNMTi treatment increases CD38 expression in MM cell lines and MM cells, and that this increase can be exploited to augment the efficacy of daratumumab-mediated lysis of MM cells.

**Fig. 1:**
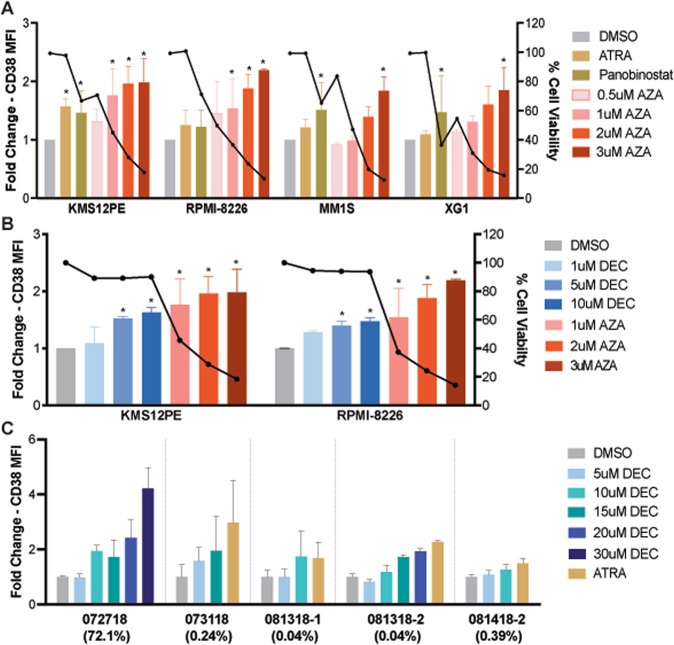
DNMTi treatment increases CD38 cell surface expression. **a** Bar graph of CD38 expression and cell survival upon treatment with different drugs in the indicated cell lines. Height of the bars indicates the fold change in CD38 MFI (left *y*-axis) and bold black line shows % cell viability (right *y*-axis). Both are normalized to DMSO treatment. Colors indicate the drug and concentration used. Data are presented as mean ± SD from 2 to 3 independent experiments with technical triplicate in each experiment. Asterisk indicates a significant difference from DMSO treatment (*p* ≤ 0.05 using a paired student’s *t*-test). For AZA treatment results are shown for 3d + 4 treatment. **b** Bar graph comparing azacytidine and decitabine treatments. As before, height of the bars indicates the fold change in CD38 MFI (left *y*-axis) and bold black line shows % cell viability (right *y*-axis). Cell viability is ~95% at 10 uM decitabine compared with ~40% for 1 uM azacytidine. Asterisk indicates a significant difference from DMSO treatment (*p* ≤ 0.05 using a paired student’s *t*-test). **c** CD38 expression on primary MM cells upon decitabine treatment. Gray lines separate different patient samples (IDs on *x*-axis). The percentage of CD138+ cells in each sample are given in brackets underneath the IDs. Data are presented as mean ± SD from three replicate wells

Detailed methods for all assays are provided in the Supplement. We treated a panel of MM cell lines (RPMI-8226, MM.1S, XG-1, and KMS12-PE) with increasing doses of AZA and analyzed CD38 cell surface expression by flow cytometry. We treated cells for either 3 or 5 days with flow cytometry analysis at 7 days (“3d + 4” or “5d + 2”, respectively). As suggested by work in other systems, this extra time is allowed for DNMTi incorporation into newly synthesized DNA in replicating cells over two doublings, leading to DNMT degradation and loss of DNA methylation. 3d + 4 and 5d + 2 treatments induced a 1.2–2.4-fold increase in CD38 MFI in a dose dependent manner for all four cell lines (Fig. S2a). 3 μM AZA consistently induced at least a twofold increase in CD38 MFI in all cell lines, in both the 3d + 4 and 5d + 2 treatments. While the 5d + 2 treatment upregulated CD38 more than 3d + 4 treatment, it also caused higher toxicities (90–98%). Hence, we used 3d + 4 for future analysis to maximize cells available for analysis and minimize cell death artefacts. Importantly, at doses >1 μM AZA appears to upregulate CD38 equally if not more than previously investigated doses of the known inducers ATRA and Panobinostat [1, 4] (Figs. 1b and S2b).

We next assessed *CD38* RNA expression in treated RPMI-8226 cells using qRT-PCR (Fig. S3a). Intriguingly, all treatments except Panobinostat appeared to induce higher fold changes in *CD38* expression at the RNA level compared with cell surface expression. This result indicates that there may be additional mechanisms regulating CD38 expression posttranscriptionally. Furthermore, our findings suggest that Panobinostat may specifically affect CD38 through a nonepigenetic mechanism. Several HDAC inhibitors indeed deacetylate nonhistone proteins [9] and recent work indicated that the surface marker CD20 is regulated translationally, not transcriptionally, after HDACi treatment in a lymphoma model [10].

To confirm our results, we repeated our experiments with DEC. DEC treatment induced similar upregulation in CD38 cell surface expression while causing minimal toxicity (2–10%) (Fig. 1b). This finding demonstrates that increased surface CD38 found after DNMTi is not caused by selective cell death of CD38-low cells.

We next treated primary myeloma cells (*n* *=* 5) with increasing doses of AZA (Fig. S3b) and DEC (Fig. 1c), using ATRA as a positive control. In contrast to our findings in MM cell lines, ATRA induced greater upregulation of CD38 than both DNMTis. This result is expected since primary MM plasma cells do not typically proliferate ex vivo, thereby preventing DNMTi incorporation during DNA synthesis and largely negating DNMTi mechanism of action. In contrast, MM patients have continually proliferating plasma cells in the marrow. We therefore expect that DNMTi treatment will lead to effective increases in CD38 cell surface expression when used in vivo in patients, though confirmation will await clinical trials. Higher upregulation by DEC compared with AZA might be attributed to lower toxicities and slightly different mechanisms [7].

Since improved chromatin accessibility induced by hypomethylation can allow increased transcription factor binding, we tested whether DNMT inhibition would show additive effects with ATRA treatment in upregulating CD38 expression. Indeed, we found increased surface CD38 upregulation using the combination of either AZA or DEC with ATRA (Fig. S4a, b). These results underscore the potential clinical utility of combining DNMTi + ATRA with daratumumab.

**Fig. 2:**
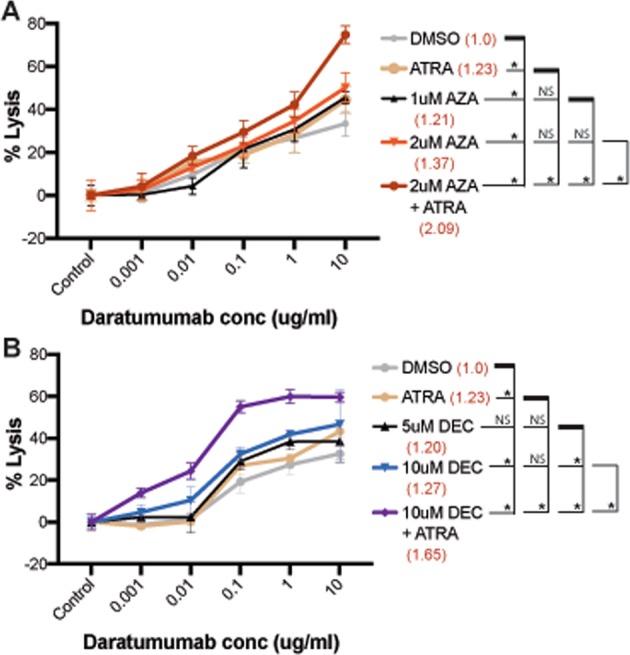
DNMTi treatment shows additive effect with ATRA in ADCC assays. Results from ADCC assays with azacytidine (**a**) and decitabine (**b**). Cell lysis is depicted on the *y*-axis with increasing concentrations of daratumumab (*x*-axis). The percent lysis by ADCC was calculated as outlined in the Methods section. Colors indicate the treatments, and fold change in CD38 MFI are given in brackets (red). Depicted is one representative experiment from two independent experiments. The *p*-values at 10 ug/ml concentration are given below in the table. Asterisk indicates a significant difference (*p* ≤ 0.05) and NS indicates *p* > 0.05 (paired student’s *t*-test)

Next, we determined whether upregulated CD38 MFIs increased functional daratumumab efficacy. Using an immortalized transgenic NK cell line to mediate lysis, we observed a significant increase in antibody dependent cellular cytotoxicity (ADCC) against DNMTi treated vs. control (Fig. 2a, b). Importantly, this increase in ADCC was consistent with the level of CD38 MFI upregulation. Furthermore, the additive effect of DNMTi and ATRA on CD38 upregulation was also seen in ADCC assays. 2 μM AZA + ATRA increased CD38 MFI 2.01 ± 0.03-fold and increased ADCC lysis to 74.77 ± 4.04%. Similarly, combination treatment with DEC and ATRA showed additive effects on CD38 upregulation and ADCC with daratumumab.

Surprisingly, targeted bisulfite sequencing of the *CD38* CpG island revealed almost complete hypomethylation at baseline in RPMI-8226 and KMS12-PE cells (Fig. S5). We therefore hypothesized that DNMTi treatment might instead upregulate CD38 indirectly. ENCODE ChIP-seq data suggests the transcription factors PU.1 and ATF2 may regulate CD38 transcription, and we therefore tested whether AZA upregulates CD38 via PU.1 or ATF2. However, knockdown of these genes did not modulate AZA-induced CD38 upregulation (Fig. S6a, b), suggesting AZA does not regulate CD38 via either of these transcription factors. Next, we tested whether activation of interferon response, a known effect of DNMTi due to derepression of endogenous retrovirus expression [11], might upregulate CD38. However, AZA-induced CD38 upregulation was not blocked by a neutralizing antibody to interferon receptor (Fig. S6c), suggesting other mechanisms are involved.

We therefore tested whether DNMTi might function via TNFα upregulation. TNFα is regulated by DNA methylation [12] and is known to upregulate CD38 expression in airway smooth muscle cells [13]. Cotreatment with a TNFα-neutralizing antibody completely abrogated AZA-induction of CD38 upregulation (Fig. S7a). Furthermore, AZA treatment indeed induced TNFα secretion from RPMI-8226 cells (Fig. S7b). Finally, recombinant TNFα increased surface CD38 in RPMI-8226 cells (Fig. S7c). Our results therefore confirm that indirect mechanisms can mediate DNMTi-induced CD38 upregulation and suggest the TNFα pathway may play a leading role in this process.

In summary, our results demonstrate that DNMTis induce a significant increase in CD38 expression on MM plasma cells. Importantly, we show that this DNMTi-induced increase in CD38 expression can be exploited to enhance the anti-MM efficacy of daratumumab through increased ADCC. DNMTi’s are used in other hematological cancers for their antitumor activity and are being investigated in MM in combination with standard therapies (NCT01155583, NCT01050790), though a prior single-agent AZA study showed little effect (NCT00412919). Notably, treatment with DNMTi is generally well tolerated and pharmacokinetic data indicates that blood plasma levels reach the concentrations we test here in vitro [14, 15]. Clinical trials are currently ongoing to investigate the combination of another well-tolerated but noncytotoxic agent, ATRA, to enhance daratumumab efficacy via increased CD38 expression (NCT02751255). Therefore, even in the case of limited direct MM cytotoxicity by DNMTi, we believe our data warrant clinical trials to investigate the safety and efficacy of repurposing DNMTi in combination with daratumumab, and potentially the DNMTi + ATRA + daratumumab combination. Potential roles could either be in the context of enhancing daratumumab therapy at first administration or in the context of attempted reversal of daratumumab resistance.

## Supplementary information

Supplementary Methods

CpG island, DNA methylation and expression of CD38 gene

Azacytidine treatment increases CD38 cell surface expression

Azacytidine upregulates CD38 transcript expression

DNMTi treatment shows additive effect with ATRA on CD38 upregulation

CD38 CpG methylation at baseline

AZA induces CD38 upregulation independently of IFN, PU.1 and ATF2

AZA induces CD38 upregulation via TNFα upregulation
