# Supplementary material for: DNA methyltransferase inhibitors upregulate CD38 protein expression and enhance daratumumab efficacy in multiple myeloma
Source: Leukemia. 2019 Oct 8;34(3):938–41. doi: 10.1038/s41375-019-0587-5 (PMC7214267; doi:10.1038/s41375-019-0587-5)
Supplement: Supplementary file 1 — Supplementary Methods [file 41375_2019_587_MOESM1_ESM.docx]

**Supplementary Methods:**

**Human Subjects**

Bone marrow aspirate samples were obtained from patients after written informed consent to participate in research protocols approved by the Institutional Review Board of the University of California, San Francisco (UCSF) and Multiple Myeloma Translational Initiative (MMTI).

**Drug treatment of cell lines**

MM cell lines (RPMI-8226, MM.1S, XG1, KMS12PE) were cultured in RPMI-1640 supplemented with 10% fetal bovine serum (Gemini). Cells were treated with addition of freshly prepared azacytidine (Sigma) in fresh media every day of the treatment. Cells were either treated continuously for three days (3d) and allowed to recover for four days in fresh media (3d+4), or they were treated for five days (two doublings) and allowed to recover for two days (5d+2). All results shown here for AZA are from lot number SLBT3630. AZA powder was always stored under Argon for long term stability. We observed lot to lot differences in CD38 upregulation with AZA. Lot number SLBS1417V induced a much higher upregulation of CD38, and higher viability on 3d+4 compared to lot number SLBT3630. However, we did not have enough material to complete three replicates for all cell lines with lot SLBS1417V and data is not shown here for that lot. 10nM ATRA (Sigma) and 10nM Panobinostat (Selleck Chemicals) were used as positive controls. For ATRA, panobinostat and decitabine (Fisher Scientific), appropriate volume of stocks was added once, and cells were assessed by flow cytometry 3 days post treatment. Stocks for ATRA were stored at -80­^o^C as per manufacturers recommendations.

**Combination drug treatment of MM cell lines**

For combination drug treatments we modified the 3d+4 regiment to allow ATRA treatment. We treated KMS12PE cells with AZA for 3 days, let them recover for 2 days, and then treated with ATRA for the last 2 days. DMSO was used as control for both AZA (DMSO +ATRA), and ATRA (2uM AZA + DMSO). For example, for DMSO + ATRA cells were treated with fresh media and DMSO for 3 days (instead of AZA), allowed to recover for 2 days, and then treated with ATRA for 2 days. For decitabine combination treatment cells were first treated with decitabine alone for 3 days, then treated with both decitabine and ATRA for 2 days.

**Drug treatment of patient samples**

We separated mononuclear cells from MM patient bone marrow aspirates by density gradient centrifugation using Histopaque (Sigma). Cells were resuspended in RPMI-1640 medium supplemented with 20% FBS and 2x10e5 cells were added to each well of a U-bottom 96-well plate. Cells were treated with increasing concentration of DNMTis *ex vivo* for 3 days and analyzed by flow cytometry.

**Flow Analysis**

MM Cell lines were stained with APC-conjugated CD38 antibody (BD 555462). SYTOX Green was used to discriminate live/dead cells. Staining was assessed on CytoFLEX flow cytometer (Beckman Coulter). FlowJo (FlowJo, LLC) was used to analyze cytotoxicity and measure Median Fluorescence Intensity (MFI) of the live cells. Changes in CD38 cell surface expression were reported as fold change in MFI as compared to DMSO control. Percent cell survival was calculated by normalizing percentage of live cells in drug-treated with DMSO-treated samples.

We stained treated patient samples with APC-R700-conjugated CD138 antibody (BD 566050), FITC-conjugated CD38 antibody (BD 555462) and PI. We assessed CD38 expression in the CD138 positive population using FlowJo software.

**RNA analysis**

RNA was extracted from treated RPMI-8226 cells using RNeasy kits (Qiagen). We performed qRT-PCR for *CD38* mRNA using primers: F: 5’- AGACTGCCAAAGTGTATGGGA-3’ and R: 5’- GCAAGGTACGGTCTGAGTTCC-3’ and SsoAdvanced SYBR Green Supermix (BioRad) on a StepOnePlus machine (Applied Biosystems). GUSB was used as a housekeeping gene (F: 5’-AGCCAGTTCCTCATCAATGG-3’ R: 5’-GGTAGTGGCTGGTACGGAAA-3’).

**Antibody-dependent cellular cytotoxicity (ADCC) assay**

Post drug-treatment of KMS12PE cells, we separated live cells from the dead cells using Histopaque density-gradient centrifugation. While 3 μM AZA upregulated CD38 expression the most, it was very cytotoxic. To obtain live cells for ADCC assays we instead used 2 μM AZA. Live cells were incubated for one hour with IgG1 isotype (BD) or increasing concentrations of daratumumab in quadruplicate wells of a white, opaque 384 well plate (Corning). Daratumumab was sourced from Janssen Pharmaceuticals. NK92-CD16 transgenic cells (a kind gift of Dr. Bruce Walchek, University of Minnesota) were then added an effector-to-target ratio of 20:1. After 20 hours, lysis was measured in a bioluminescence-based assay using CytoToxGlo (Promega). Lysis by ADCC was calculated using the following formula : % Lysis = (signal in presence of daratumumab – signal in presence of IgG1 control antibody) x100 / signal in presence of IgG1 control antibody.

**Array-based methylation analysis using HELP**

DNA methylation data for MM patients (*n*=31) and plasma cells from normal individuals (*n*=8) were obtained from the GEO database accession number GSE43860, which was on the HELP (HpaII tiny fragment enrichment by ligation mediated PCR) human HG_17 promoter custom-designed oligonucleotide methylation array by NimbleGen. HELP array covers 25,626 HpaII amplifiable fragments annotated to 14,214 gene promoters. DNA was digested with isoschizomer enzymes HpaII (for unmethylated CpG) and MspI (for both methylated and unmethylated CpG) and co-hybridized to HELP array. The array data for each channel (HpaII or MspI) were generated using NimbleScan software, and represented as signal intensities at each HpaII amplifiable fragment, which were calculated as a robust (25% trimmed) mean of their component probe-level signal intensities. The log_2_-ratio (HpaII/MspI) was calculated for each locus, where a relatively high value of log_2_(HpaII/MspI) for hypo-methylation and a low value for hyper-methylation. A median normalization was performed on each array by subtracting the median log-ratio (HpaII/MspI) of that array (resulting in median log-ratio of 0 for each array). HELP data analysis was performed as described previously(1), using R software (R Core Team 2013) and Bioconductor package (http://www.bioconductor.org/). The differential methylation between two groups of samples was calculated with two-sided *t*-test in R.

**Expression microarray data**

Gene expression microarray data was obtained from the GEO database accession number GSE17306 and GSE12453. The expression microarray was on the GeneChip Human Genome U133 Plus 2.0 Array by Affymetrix. It contains 5 normal plasma cell samples and 52 MM patient samples. The raw CEL data were processed and normalized using the MAS5 method with the Expression Console software (Affymetrix). The value for each probeset was represented as log*_2_* signal intensity from the array. The differential expression between two groups of samples was calculated with two-sided t-test in R.

**Targeted Bisulfite Sequencing**

RPMI-8226 and KMS12PE cells were treated with DMSO or 3uM AZA and genomic DNA was extracted using Quick-DNA kit and Genomic DNA Clean and Concentrator kits (Zymo Research). Samples were shipped to Zymo Research for bisulfite conversion, multiplex amplification, barcoding and paired-end sequencing. Sequence reads were identified using standard Illumina base-calling software and then analyzed using a Zymo Research proprietary analysis pipeline. Sequence reads were aligned back to the reference genome using Bismark (http://www.bioinformatics.babraham.ac.uk/projects/bismark/), an aligner optimized for bisulfite sequence data and methylation calling(2). The methylation ratio of each sampled cytosine was estimated as the number of reads reporting a C, divided by the total number of reads reporting a C or T.

**Combination treatment with Cytokine Blocking and AZA**

For combination AZA-cytokine blocking treatments we modified the 3d+4 regimen. We treated RPMI-8226 cells with fresh media containing the respective antibody and either DMSO or 3 μM AZA consecutively for 3 days, and then changed to media with antibody only on day 4. Cells were allowed to recover as before and CD38 cell surface expression was measured at 7 days. 0.5 μg/ml of Rabbit IgG1 (Fisher Scientific, 026102) was used as control. For blocking interferon signaling 0.5 μg/ml (= 5.6 neutralization Units/ml) of neutralizing anti-IFNR antibody (PBL assay science, #21385-1) was added. Similarly, for blocking TNFα we added 10 ng/ml of a rabbit human TNFα neutralizing antibody (Cell Signaling Technologies, #7321S).

**Knockdown of Transcription factors using CRISPR interference**

RPMI-8266 cell line expressing dCas9/KRAB fusion proteins(3) was a kind gift from Dr Martin Kampmann (UCSF). Oligonucleotides encoding sgRNAs against PU.1 and ATF2 were cloned into lentiviral vector for expression from a U6 promoter. Lentivirus were generated using LentiX cells. RPMI-8226 dCAS9/KRAB cell line was transduced, and clones were obtained by puromycin selection.

**TNFα treatment and ELISA**

TNFα (BD Pharmingen) was added to RPMI-8226 cells exogenously at the indicated concentrations and CD38 cell surface expression measured by flow after 72 hours. For ELISA RPMI-8226 were treated as per the 3d+4 regimen, and on day 7 live cells were counted with trypan blue, centrifuged and CD38 upregulation confirmed by flow. The supernatant was collected, aliquoted and frozen at -80 ^o^C for future analysis. TNFα ELISA kit (Sigma, RAB0476) with the standards was used to measure levels of secreted TNFα in the frozen supernatants.

**Supplementary References**

1. Geng H, Brennan S, Milne TA, Chen WY, Li Y, Hurtz C, et al. Integrative epigenomic analysis identifies biomarkers and therapeutic targets in adult B-acute lymphoblastic leukemia. Cancer Discov. 2012;2(11):1004-23.

2. Krueger F, Andrews SR. Bismark: a flexible aligner and methylation caller for Bisulfite-Seq applications. Bioinformatics. 2011;27(11):1571-2.

3. Gilbert LA, Horlbeck MA, Adamson B, Villalta JE, Chen Y, Whitehead EH, et al. Genome-Scale CRISPR-Mediated Control of Gene Repression and Activation. Cell. 2014;159(3):647-61.

**Supplementary Figure Legends**

**Fig. S1: CpG island, DNA methylation and expression of *CD38* gene.** (A) Increasingly zoomed images of the *CD38* locus (modified from UCSC browser) showing the locations of CpG island, exon 1, intron 1 and putative promoter. Dotted grey line shows the position of the Transcription Start Site (TSS). (B) DNA methylation from GSE43860 and (C) RNA expression from GSE17306 and GSE12453 for *CD38*. * indicates a significant difference (*p* ≤ 0.05).

**Fig. S2: Azacytidine treatment increases CD38 cell surface expression**. (A) Bar graph of CD38 expression and cell survival upon treatment with azacytidine in the indicated cell lines. For each cell line the 3d, 3d+4 and 5d+2 treatments are compared side-by-side. Height of the bars indicates the fold change in CD38 MFI (left y-axis) and bold black line shows % cell viability (right *y*-axis). Both are normalized to DMSO treatment. Colors indicate the concentration of azacytidine used. Data are presented as mean ­± SD from 2-3 independent experiments with triplicates in each experiment. * indicates a significant difference from DMSO treatment (*p* ≤ 0.05 using a paired student’s *t*-test). (B) shows the histogram plots for CD38 expression for one representative experiment in XG1 cells. Histograms from 3d+4 azacytidine treatment are compared to the known CD38 upregulators ATRA and Panobinostat. The drugs used are indicated on the left, and fold change in CD38 MFI compared to DMSO control is given on the right. The grey dotted line indicates MFI in DMSO treated cells.

**Fig. S3: Azacytidine upregulates *CD38* transcript expression**. (A) Bar graph showing fold change in CD38 RNA (red) and cell surface protein (blue) in RPMI-8226 cells upon drug treatments. Data from 3d+4 and 5d+2 azacytidine treatments are grouped together and compared to ATRA and panobinostat treatment. Data is presented as mean ± SD from a representative experiment from two independent experiments. (B) CD38 expression on primary MM cells upon azacytidine treatment. Grey lines separate different patient samples (IDs on *x*-axis). Colors in the legend indicate the drug and concentration used. The percentage of CD138+ cells in each sample are given in brackets underneath the IDs. Data are presented as mean ­± SD from 3 replicate wells.

**Fig. S4: DNMTi treatment shows additive effect with ATRA on CD38 upregulation.** Upregulation of CD38 MFI upon combination treatment with ATRA and azacytidine (A) or decitabine (B). Combination treatments were done as described in the methods section. X, Y and Z represent independent experiments, and data are presented as mean ­± SD from triplicate wells. While the level of CD38 upregulation is different in each experiment, DNMTi treatment consistently induces upregulation of CD38 that is additive with ATRA-induced upregulation of CD38. * indicates a significant difference (*p* ≤ 0.05 using a paired student’s *t*-test).

**Fig. S5: *CD38* CpG methylation at baseline.** Targeted Bisulfite seq data visualized in UCSC browser. *CD38* RefSeq RNAs and CpG island are depicted in blue and green respectively. The CpG Methylation track highlights each CpG site as a vertical bar. The label on the left of each CpG bar indicates the methylation ratio followed by percent methylation in brackets. For example, the first CpG labeled ‘4/6932 (0%)’ indicates 4 reads were methylated in 6932-fold sequencing read depth of coverage at this site, amounting to 0% methylation ratio. Data for RPMI-8226 is depicted here as an example. KMS12PE cell line was similarly completely hypomethylated.

**Fig. S6: AZA induces CD38 upregulation independently of IFN, PU.1 and ATF2.** RPMI-8226 line with an integrated dCas9-KRAB construct was transduced with a plasmid carrying scrambled sgRNA (Scr-i) or two separate guides against PU.1 (A) and ATF2 (B). Data are represented as Fold Change in CD38 cell surface expression upon 3 μM AZA treatment normalized to DMSO treatment for each cell line. In these results, knockdown of both transcription factors could not prevent Aza-induced upregulation of CD38. (C) RPMI-8266 were co-treated with 3 μM AZA treatment and either IgG1 (control) or neutralizing anti-IFNR antibody (αIFNR). CD38 cell surface expression upon 3 μM AZA treatment is normalized to DMSO treatment for the same Antibody treatment. αIFNR does not lead to consistent blockade of CD38 induction after Aza.

X and Y represent independent experiments, and data are presented as mean ­± SD from triplicate wells. * indicates a significant difference (*p* ≤ 0.05 using a paired student’s *t*-test), while NS indicates *p* > 0.05.

**Fig. S7: AZA induces CD38 upregulation via TNFα upregulation.** (A) RPMI-8266 were co-treated with 3 μM AZA treatment and either IgG1 (control) or a neutralizing anti-TNFα antibody (αTNFα). Data are represented as Fold Change in CD38 cell surface expression upon 3 μM AZA treatment normalized to DMSO treatment for the same Antibody treatment. αTNFα completely blocks surface induction of CD38 after Aza treatment. * indicates a significant difference between CD38 Fold change in IgG1- versus αTNFα- treated cells in individual experiments (*p* ≤ 0.05 using a paired student’s *t*-test). (B) TNFα concentrations were measured in supernatants from RPMI-8266 cell line after Aza treatment. Data are represented as fold change in TNFα concentration compared to DMSO. X and Y represent independent experiments, and data are presented as mean ­± SD from triplicate wells. X-norm and Y-norm are data from independent replicate experiments X and Y normalized to the live cell concentration measured at the time of supernatant collection. * indicates a significant difference between values from 3 μM AZA treatment and DMSO control (*p* ≤ 0.05 using a paired student’s *t*-test). (C) Fold change in CD38 cell surface expression after 72-hour treatment of RPMI-8226 cells with exogenously added recombinant TNFα. Data are represented as mean ± SEM of two independent experiments. * indicates a significant difference (*p* ≤ 0.05 using a paired student’s *t*-test).
