# Supplementary figures and images for: DNA methyltransferase inhibitors upregulate CD38 protein expression and enhance daratumumab efficacy in multiple myeloma
Source: Leukemia. 2019 Oct 8;34(3):938–41. doi: 10.1038/s41375-019-0587-5 (PMC7214267; doi:10.1038/s41375-019-0587-5)

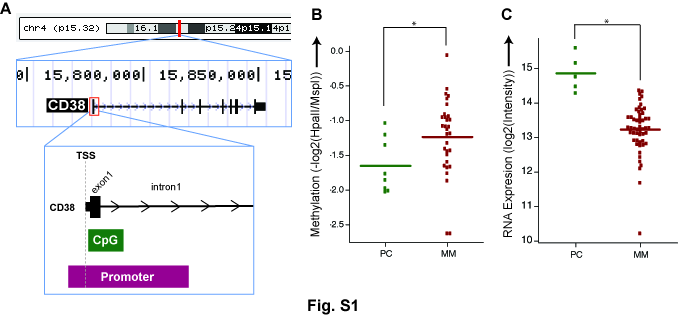

Supplement: Supplementary file 2 — CpG island, DNA methylation and expression of CD38 gene [file 41375_2019_587_MOESM2_ESM.tif]

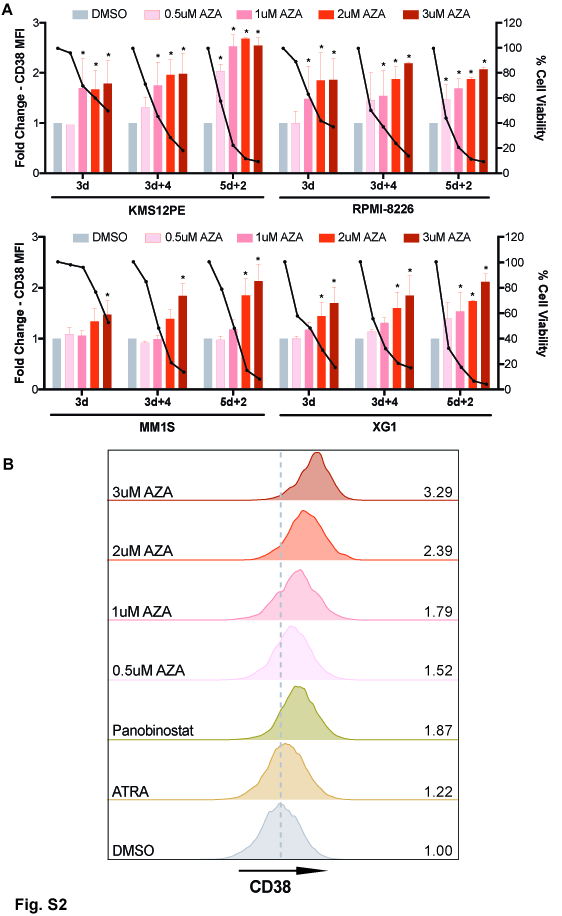

Supplement: Supplementary file 3 — Azacytidine treatment increases CD38 cell surface expression [file 41375_2019_587_MOESM3_ESM.tif]

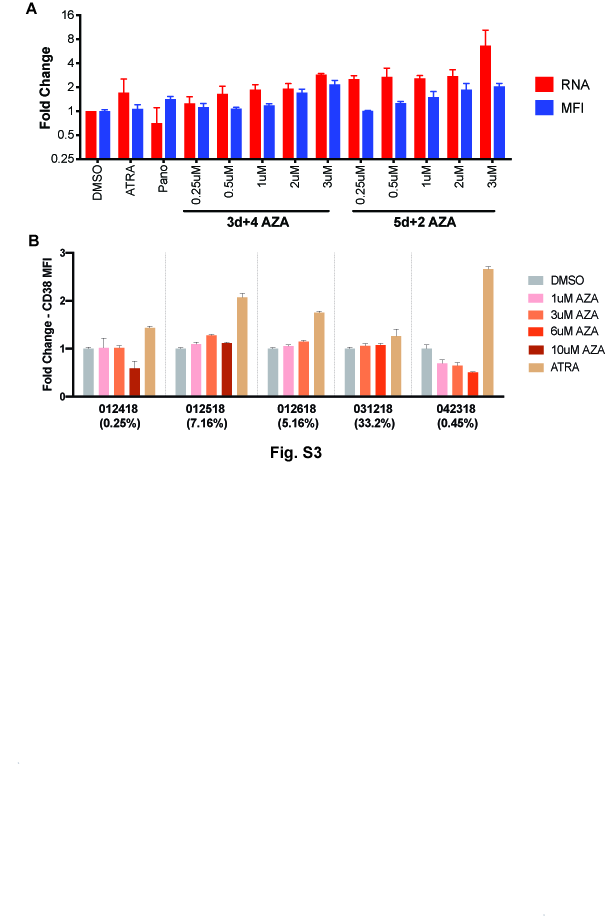

Supplement: Supplementary file 4 — Azacytidine upregulates CD38 transcript expression [file 41375_2019_587_MOESM4_ESM.tif]

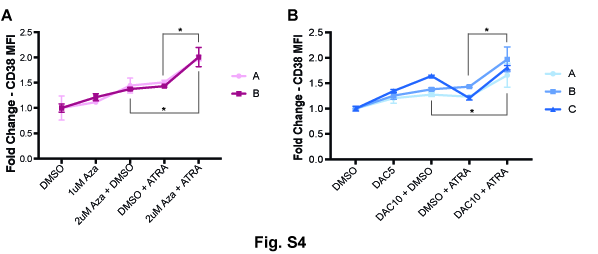

Supplement: Supplementary file 5 — DNMTi treatment shows additive effect with ATRA on CD38 upregulation [file 41375_2019_587_MOESM5_ESM.tif]

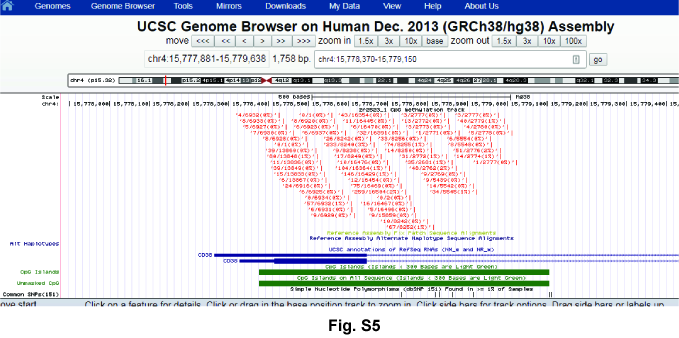

Supplement: Supplementary file 6 — CD38 CpG methylation at baseline [file 41375_2019_587_MOESM6_ESM.tif]

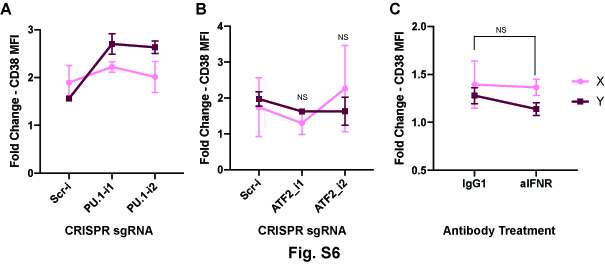

Supplement: Supplementary file 7 — AZA induces CD38 upregulation independently of IFN, PU.1 and ATF2 [file 41375_2019_587_MOESM7_ESM.tif]

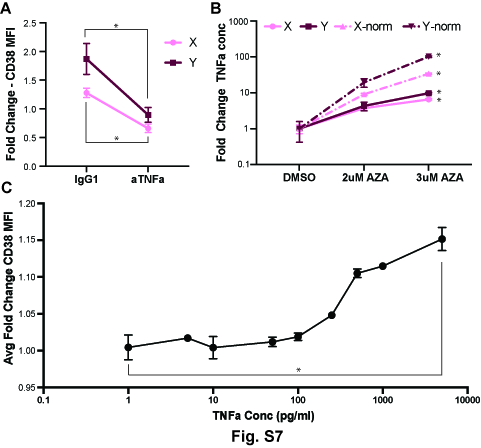

Supplement: Supplementary file 8 — AZA induces CD38 upregulation via TNFα upregulation [file 41375_2019_587_MOESM8_ESM.tif]
